# Lab-on-a-Chip Platforms for Detection of Cardiovascular Disease and Cancer Biomarkers

**DOI:** 10.3390/s17122934

**Published:** 2017-12-17

**Authors:** Jiandong Wu, Meili Dong, Susy Santos, Claudio Rigatto, Yong Liu, Francis Lin

**Affiliations:** 1 Department of Physics and Astronomy, University of Manitoba, Winnipeg, MB, R3T 2N2, Canada; wujd@physics.umanitoba.ca (J.W.); dongmeili@aiofm.ac.cn (M.D.); 2 Institute of Applied Technology, Hefei Institutes of Physical Science, Chinese Academy of Sciences, Hefei 230088, China; 3 Victoria General Hospital and River Heights/Fort Garry Community Areas, Winnipeg, MB, R3T 2E8, Canada; ssantos3@vgh.mb.ca; 4 Seven Oaks General Hospital, Winnipeg, MB, R2V 3M3, Canada; CRIGATTO@sbgh.mb.ca

**Keywords:** lab-on-a-chip, cardiovascular disease, cancer, biomarker, microfluidic, point-of-care

## Abstract

Cardiovascular disease (CVD) and cancer are two leading causes of death worldwide. CVD and cancer share risk factors such as obesity and diabetes mellitus and have common diagnostic biomarkers such as interleukin-6 and C-reactive protein. Thus, timely and accurate diagnosis of these two correlated diseases is of high interest to both the research and healthcare communities. Most conventional methods for CVD and cancer biomarker detection such as microwell plate-based immunoassay and polymerase chain reaction often suffer from high costs, low test speeds, and complicated procedures. Recently, lab-on-a-chip (LoC)-based platforms have been increasingly developed for CVD and cancer biomarker sensing and analysis using various molecular and cell-based diagnostic biomarkers. These new platforms not only enable better sample preparation, chemical manipulation and reaction, high-throughput and portability, but also provide attractive features such as label-free detection and improved sensitivity due to the integration of various novel detection techniques. These features effectively improve the diagnostic test speed and simplify the detection procedure. In addition, microfluidic cell assays and organ-on-chip models offer new potential approaches for CVD and cancer diagnosis. Here we provide a mini-review focusing on recent development of LoC-based methods for CVD and cancer diagnostic biomarker measurements, and our perspectives of the challenges, opportunities and future directions.

## 1. Introduction

**Figure 1:**
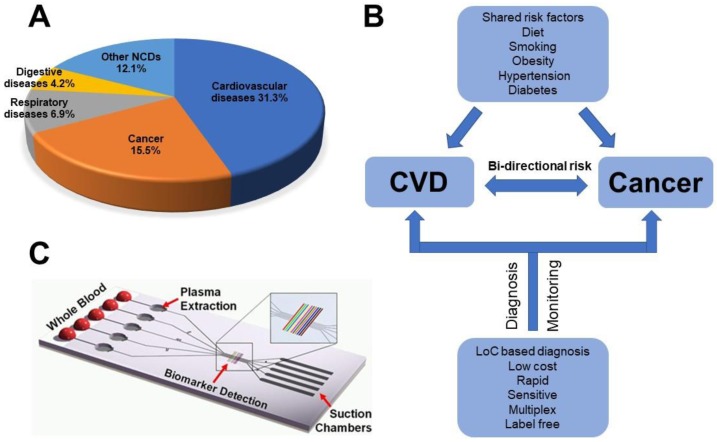
Lab-on-a-chip (LoC) platforms for cardiovascular disease (CVD) and cancer biomarkers detection. (**A**) CVD and cancer are leading causes of death worldwide according to WHO (the pie chart only shows noncommunicable diseases (NCDs)); (**B**) Shared risk factors between CVD and cancer and LoC platform can improve disease diagnosis and monitoring; (**C**) An example of LoC-based protein biomarker detection from blood [2]. Figure C is adapted from Ref. 2 with permission of The Royal Society of Chemistry.

According to the World Health Organization (WHO), noncommunicable diseases (NCDs) were responsible for 70% of all deaths worldwide in 2015. Cardiovascular disease (CVD) and cancer, the two major types of NCDs, account for two-thirds of global NCDs deaths (Figure 1A). Mounting evidence shows that these two diseases share several risk factors including diet, tobacco smoking, hypertension, diabetes and obesity, suggesting some common genetic and molecular mechanisms [1]. For example, both CVD and cancer are correlated with chronic inflammation. Atherosclerosis is a chronic inflammatory disease of the arterial wall, leading to myocardial infarction, stroke, and peripheral vascular disease. In most solid tumors, malignant cells interact in a complex, chronically inflamed extracellular microenvironment, which is enriched with macrophages, inflammatory cytokines, growth factors, and reactive oxygen species. These shared biological mechanisms between CVD and cancer naturally suggest some common diagnostic biomarkers (Figure 1B), such as the elevation of C-reactive protein (CRP), which is a biomarker for inflammation. Rapid and early disease diagnosis gives hope for improved treatment and the control of disease progression. In this regard, molecular quantitation of specific protein or genetic biomarkers in blood or other physiological fluids such as urine or saliva is typically required. However, traditional biomarker detection methods such as enzyme linked immunosorbent assay (ELISA) and polymerase chain reaction (PCR) heavily rely on sophisticated, bulky, and expensive equipment in centralized laboratories. Thus, researchers have been motivated to develop new diagnostic tools suitable for rapid point-of-care (PoC) applications.

Over the past two decades, lab-on-a-chip (LoC) technology has drawn significant interest from the research communities and industries for biomedical applications, owing to the advantages of LoC platforms in biological sample processing, high throughput, low reagent and sample consumption, short assay time, and multiplexed detection [3,4,5,6,7,8]. In particular, the LoC technology has shown potential to improve molecular biomarker detection by offering sensitive and wide-ranging measurements in a compact format (Figure 1C). More recently, LoC technology has been applied to studying cell-based disease biomarkers and organ-on-chip models [9]. The development of heart-on-chip and cancer-on-chip models was envisioned to be useful tools to better understand CVD risk and cancer metastasis. Some excellent earlier review papers are available on biosensors for CVD and cancer. They focus on specific technologies such as electrochemical detection [10], immunoassay [11], and cancer-on-chip [12]. In this mini review, we focus on recent developments of different LoC-based methods for the measurements of different CVD and cancer diagnostic biomarkers, including proteins, nucleic acids, and cells. This review is organized to first introduce the general background of CVD and cancer diagnosis and relevant LoC technologies, which will be followed by a detailed review of LoC-based diagnosis applications for each disease, and concluded by discussing our perspectives of the challenges, opportunities, and future directions.

## 2. LoC Platforms for Detection of Cardiovascular Disease Biomarkers

**Figure 2:**
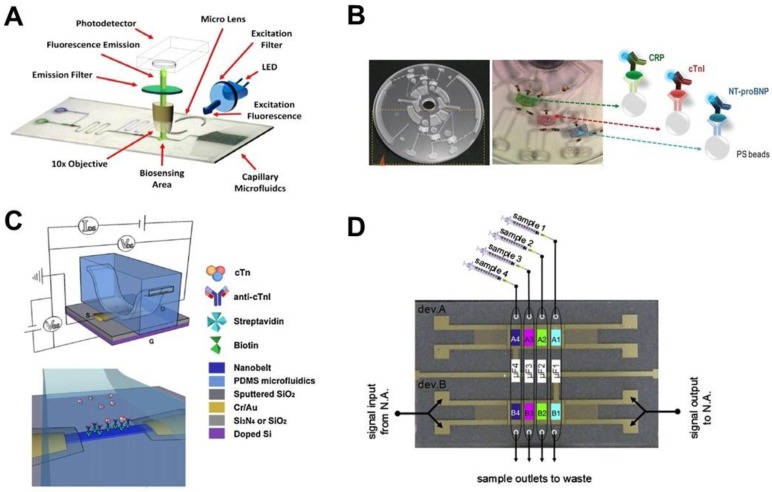
Examples of LoC-based platforms for CVD biomarker detection. (**A**) An autonomous capillary microfluidic platform with embedded optics for troponin I detection [31]; (**B**) a lab-on-a-disc platform for fully integrated multiplex cardiac marker immunoassays [16]; (**C**) functionalized SnO_2_ nanobelt field-effect transistor sensors for label-free detection of cardiac troponin [32]; (**D**) detection of multiple cardiac markers with an integrated acoustic platform for cardiovascular risk assessment [18]. Figure A is reprinted from Mohammed et al., Autonomous capillary microfluidic system with embedded optics for improved troponin I cardiac biomarker detection, Biosens. Bioelectron., 61, 478–484, Copyright (2014), with permission from Elsevier; Figure B is reprinted with permission from Park et al., Lab-on-a-Disc for Fully Integrated Multiplex Immunoassays. Anal. Chem. 2012, 84, (5), 2133–2140. Copyright (2012) American Chemical Society; Figure C is reprinted from Cheng et al., Functionalized SnO_2_ nanobelt field-effect transistor sensors for label-free detection of cardiac troponin, Biosens. Bioelectron., 26, 4538–4544, Copyright (2011), with permission from Elsevier; Figure D is reprinted from Mitsakakis et al., Detection of multiple cardiac markers with an integrated acoustic platform for cardiovascular risk assessment, Anal. Chim. Acta, 699, 1–5, Copyright (2011), with permission from Elsevier.

CVD caused 17.3 million deaths in 2013 compared to 12.3 million in 1990 [13], of which an estimated 90% are thought to have been preventable [14]. However, over 50% of all cardiovascular related diseases do not present early symptoms. Among the various diagnosis tests (e.g., blood tests, electrocardiogram, stress testing, echocardiography, coronary angiography and cardiac catheterization, chest X-ray, electron-beam computed tomography and cardiac magnetic resonance imaging [MRI]), blood tests can detect different blood biomarkers, which are useful to assess the risk factors for CVD. These blood biomarkers include fats, cholesterol, lipid components, protein biomarkers for inflammation (e.g., CRP, Apolipoprotein A1) and heart attack (e.g., cardiac troponin [cTn], fibrinogen, plasminogen activator inhibitor-1 [PAI-1]). For example, cTn is a biomarker for assessing both acute and chronic myocardial injury. In the acute condition, clinical cTn tests need to meet the requirements of fast turn-around and high accuracy. In this regard, some PoC tests are already available for fast cTn detection [15]. Those tests are based on rapid immunoassays that are able to detect cTn within 30 min, and require minimal sample preparation and handling. A similar situation applies to the detection of other CVD biomarkers. The LoC-based methods have the potential to further reduce the detection time, improve the accuracy and allow simultaneous detection of multiple biomarkers. In this section, we review some representative LoC-based methods for CVD diagnosis with the focus on different immunoassays and cell-based assays (Table 1; Figure 2).

### 2.1. Detection of Protein Biomarkers

As a natural approach, standard format immunoassays have been translated to microfluidic platforms for detecting CVD biomarkers [33,34]. An immunoassay is a method to measures the concentration of small molecules by the specific binding between antibodies and antigens. The standard immunoassays often require multiple steps involving adding different capture, blocking, and detection reagents, as well as washing buffers at different time points. For example, Mohammed et al. developed an automated biosensing platform to detect cTnI. This platform integrated autonomous capillary microfluidics for passive sample and reagent transport and optical lenses for signal detection (Figure 2A) [31], of which both components are engraved using a CO_2_ laser. The detection is based on a fluorescent sandwich immunoassay, has an assay limit of detection (LOD) of 24 pg/mL, and a typical test is completed within 7–9 min. In another study, Park et al. took advantage of a lab-on-a-disc microfluidic platform to realize automatic control for cTn detection in raw samples including blood and saliva (Figure 2B) [16]. The device used centrifugal force to transfer liquid samples and the detection was based on a microbead-based sandwich ELISA. The beads, modified with different biomarker detection reagents, were initially preloaded into three reaction channels which can be optically actuated using ferrowax microvalves. Besides cTnI, two other important CVD biomarkers, high-sensitivity C-reactive protein (hs-CRP) and N-terminal pro-B type natriuretic peptide (NT-proBNP) can be detected simultaneously using this platform. The combined detection of multiple biomarkers is expected to improve disease risk prediction. Additionally, Blu-ray discs have been adapted as the substrates for immunoassays to test three cardiac markers proteins including myoglobin, cTnI, and CRP [17]. Moreover, the unmodified optical drive was directly used to digitally read the silver enhanced signal. In this platform, microfluidic channels were first attached on the discs to guide the reagents to multiple reaction areas and then removed before signal reading. The LOD of this platform was comparable to a conventional ELISA kit.

Instead of the standard immunoassay format, scientists modified immunoassays or integrated novel detection methods in LoC devices to simplify the detection procedures. For instance, Li et al. developed a blocking-free microfluidic heterogeneous immunoassay using a protein A functionalized polydimethylsiloxane (PDMS) microchannel [19]. This device was used to detect the concentration of CRP for the assessment of risk of CVD. This assay can perform five groups of tests in 5 min and the modified chip can maintain its functionality for up to 14 months when stored at 4 °C. Several studies developed novel microfluidic devices for label-free detection. Cheng et al. developed a real-time label-free electrical protein detection system by integrating functionalized SnO_2_ nanobelt field-effect transistors (FETs) into a microfluidic device (Figure 2C) [32]. The detection is based on the change of the electrical conductance which is induced by the specific binding of charged analytes to the antibodies immobilized on the nanobelt surfaces. This device was successfully applied to detecting the cTnI subunit within the human cardiac troponin complex. It can be used to detect most charged soluble molecules based on the specific high affinity binding partner immobilized on the nanobelt surface. Jang et al. also developed a label-free method by combined isoelectric focusing and microfluidics for CVD diagnosis. This device analyzes the dysfunctional protein by measuring the unique isoelectric point (pI), at which molecules have a zero net charge [35]. It was found that high density lipoprotein-3 (HDL3) isolated from the sera of elderly males has higher mobility with a broader range and a higher pI (~8.1) than from the younger male age group, which showed a narrow band range and a lower pI (~6.9). Based on these findings, the healthy and diseased states can be quickly distinguished.

The surface acoustic wave (SAW) biosensor is another method for label-free and multi-analyte detection. Mitsakakis et al. integrated a SAW device with a multi-channel microfluidic module for cardiac biomarkers detection (Figure 2D) [18]. The detection mechanism is based on the change of the phase and amplitude of the acoustic wave when biochemical processes take place on the sensor surface. The phase change is associated with the amount of bound mass and the amplitude change reflects the conformation state of the bound molecules or viscoelasticity of the biofilm. Four cardiac markers including creatine kinase MB (CK-MB), CRP, D-dimer, and pregnancy-associated plasma protein A (PAPP-A) can be detected in 30 min. The dynamic range of this device spanned two orders of magnitude covering the pathological concentration range. Kim et al. developed a polyester tomer microchip for cleavable tag immunoassay (CTI). In this assay, the fluorescent tag was cleaved from the detection antibody after a sandwich immunoassay [36]. Afterwards, the fluorescent tag was measured by electrophoresis to indirectly indicate the concentration of multiple target antigens. Shin et al. developed a microfluidic aptamer-based electrochemical sensor for continual monitoring of the CK-MB level of a heart-on-a-chip model [37]. Because of the extremely low concentration of CK-MB, a high-sensitivity electrochemical impedance spectroscopy (EIS) based sensor was developed using aptamer-coated Au electrodes, which can measure the amount of CK-MB secreted by human embryonic stem cell-derived cardiomyocytes after exposure to the cardiotoxic drug, doxorubicin, in a dose-dependent manner.

### 2.2. Detection of Cell-Based Biomarkers

In addition to protein CVD biomarkers, scientists proposed to target biological cells as new CVD biomarkers. In this direction, LoC assays have been developed for cell tests. The white blood cell (WBC) count is a useful predictor of coronary heart disease [38]. Zhang et al. developed a paper-based vertical flow platform to quantify WBC [20]. Small pores of the paper trap the WBCs, which were pre-stained by anti-CD45 antibody conjugated gold nanoparticles. The WBC count is performed by colorimetric intensity measurement of gold nanoparticles. Using this platform, WBCs in 15 μL of blood can be analyzed to differentiate abnormal and normal cell counts. Compared with traditional WBC count methods, this technique is simple and does not require special detection equipment. Therefore, it is well suited for PoC tests in low-resource settings. Besides WBC counts, the number of circulating endothelial progenitor cells (EPC) is another useful biomarker for CVD. However, current methods for isolation of EPC are complex. Hansmann et al. developed a disposable microfluidic platform to capture and count EPC directly from whole blood using only a 200 μL blood sample [21]. The blood sample was added to the microfluidic chip with anti-CD34 antibody coated microcolumns. Captured cells were analyzed by immunofluorescent staining for stem and endothelial antigen expression. This device was also used to compare blood EPC in healthy subjects with pulmonary arterial hypertension patients. The results showed that EPC is about 50% lower in arterial hypertension patients than the healthy control subjects.

In summary, LoC-based platforms not only provide better sample preparation, chemical manipulation and reaction, and high-throughput, but also provide attractive features such as the avoidance of labels and improved sensitivity by integrating different novel detection techniques. These features effectively improve the diagnostic test speed and simplify the detection procedure. In addition, microfluidic cell assays offer new potential approaches for CVD diagnosis.

## 3. LoC Platforms for Detection of Cancer Biomarkers

**Figure 3:**
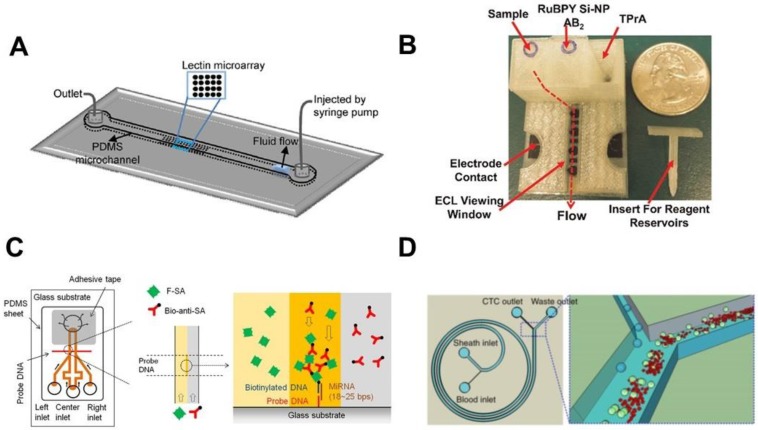
Examples of LoC-based platforms for cancer biomarkers detection. (**A**) A multiplex microfluidic platform to quantify multi-biomarkers for gastritis and gastric cancer [44]; (**B**) a 3D-printed microfluidic electrochemiluminescent (ECL) assay to detect multiple protein biomarkers for prostate cancer [22]; (**C**) a power-free microfluidic device to directly detect cancer related miRNA in a rapid and sensitive manner [24]; (**D**) a spiral microfluidic device to allow label-free size-based isolation of circulating tumor cells [30]. Figure A is reprinted from Roy et al., On-chip lectin microarray for glycoprofiling of different gastritis types and gastric cancer. Biomicrofluidics 2014, 8, (3), 034107, with the permission of AIP Publishing. Figure B is reprinted from Kadimisetty et al., 3D-printed supercapacitor-powered electrochemiluminescent protein immunoarray, Biosens. Bioelectron., 77, 188–193, Copyright (2016), with permission from Elsevier. Figure C is adapted from Ref. 24 with permission from PLOS. Figure D is reprinted by permission from Macmillan Publishers Ltd: Nature Protocols, Warkiani et al., Ultra-fast, label-free isolation of circulating tumor cells from blood using spiral microfluidics, 11, (1), 134–148, copyright (2016).

Cancer is caused by unregulated cell growth and primary tumors can metastasize to other parts of the body [39]. Cancer is among the leading causes of morbidity and mortality worldwide [40]. The most common cancers include lung, liver, stomach, colorectal, breast and oesophageal cancer [40]. Many types of cancer can be effectively treated if diagnosed early. The existing methods of cancer diagnosis rely heavily on biopsy staining, which is both invasive and may miss cancer cells at the early stage. Imaging-based tests such as mammography, CT, MRI, and sonography are available for cancer evaluation but suffer from limited resolution and expensive equipment. Various biomolecular markers for cancer diagnosis have been identified [5,41,42,43]. However, traditional methods to assess these biomarkers such as ELISA and PCR are time-consuming, expensive and often limited in detection sensitivity. LoC platforms are being increasingly developed to improve cancer biomarker detection by providing more sensitive, specific, and cost-effective testing. (Table 1; Figure 3) [23,27,28,44].

### 3.1. Detection of Protein Biomarkers

Protein biomarkers are important targets for early cancer detection and monitoring [45,46]. Particularly, a multiplexed test of a panel of protein biomarkers can improve the outcome of cancer diagnosis [5,42,47]. In this direction, Shadfan et al. configured a multiplex microfluidic platform to quantify multi-biomarkers for ovarian cancer detection [23]. The device was assembled with multiple double-sided adhesive and polyethylene layers, which were cut into fluidic channels. Agarose beads conjugated with capture antibodies were trapped in the channel to perform the sandwich immunoassays. Individual components such as membranes, glass fiber conjugate pads and mixers were integrated into the device to enable on-chip sample filtration, antibody storage, and chemical mixing. Four biomarkers with significant relevance to ovarian cancer including carbohydrate antigen 125 (CA125), human epididymis protein 4 (HE4), matrix metalloproteinase-7 (MMP-7) and Cancer Antigen 72-4 (CA72-4) were tested with this device using serum and plasma samples. This device was able to distinguish the patients encompassing early- and late-stage ovarian cancers from healthy controls with 68.7% sensitivity at 80% specificity. Glycosylation pattern changes in serum proteins and membrane proteins of tumor cells are closely associated with cancer progression [48,49,50]. The traditional technologies for glycocodes characterization such as chromatography and mass spectroscopy are limited in throughput and the ability to systematically evaluate the protein glycosylation pattern [51]. Roy et al. fabricated a microfluidic device with a microarray of 17 different lectins on the bottom surface of the channel to study the glycan structure alteration in different stages of gastritis and gastric cancer with high-throughput and low sample volume (Figure 3A) [44]. Using this platform, serum and gastric biopsy samples from normal and different type of gastritis and gastric adenocarcinoma patients were tested. Characteristic glycol-profiles were identified in the patient group but not the healthy control group. Kadimisetty et al. developed a 3D-printed microfluidic electrochemiluminescent (ECL) assay to detect multiple protein biomarkers for prostate cancer (Figure 3B) [22]. The device included a microfluidic channel with 3D printed reaction array, reagent reservoirs and washing reservoir module. The protein biomarkers were captured by the antibody-coated carbon sensor. This was followed by reaction with the Ru(bpy)_3_^2+^ (RuBPY)-doped silica nanoparticles coated with the detection-antibody in a sandwich immunoassay. The ECL signal from the electrochemical oxidation of RuBPY was captured with a CCD camera to evaluate the protein concentration. Three cancer biomarkers including prostate specific antigen (PSA), prostate specific membrane antigen (PSMA) and platelet factor-4 (PF-4) were tested and the LOD was determined to be 300–500 fg/mL in undiluted calf serum. Six prostate cancer patients’ serum samples were tested and results showed good correlation with conventional ELISA.

### 3.2. Detection of Nucleic Acid Biomarkers

Nucleic acid based measurement is also useful for cancer detection. Quantitative PCR (qPCR) is the most reliable tool for sensitive genetic quantification [52]. PCR is a technique that can amplify DNA detection by generating thousands to millions of copies of a particular DNA sequence. Quantitative PCR can monitor the amplification process in real-time by incorporating the fluorescent reporters. However, the conventional PCR machines suffer from long detection time, high sample consumption, and complex operation. LoC platforms have shown promise in addressing these limitations. For example, the digital PCR technology takes advantage of LoC-based sample partitioning using droplets or microwells and can achieve increased sensitivity and improved multiplexing capability. To explore the use of tumor-specific somatic DNA mutations as a cancer biomarkers, Azuara et al. employed a nanofluidic digital PCR to quantitatively measure the proportion of KRAS mutant alleles in colorectal and pancreatic carcinoma samples [29]. The system was able to detect six types of KRAS mutation simultaneously. Compared with other KRAS mutations detection techniques [19,53,54], this system has comparable detection sensitivity and simpler operation. Cell free circulating (cfc) DNA is another important biomarker for early detection of cancers. Sonnenberg et al. used a dielectrophoretic (DEP) microelectronic array device (although not microfluidics-based) to isolate and detect cfc-DNA in whole blood from chronic lymphocytic leukemia (CLL) patients [27]. DEP is a phenomenon that the dielectric particles can experience a force when exposed to a non-uniform electric field; it can be used to separate particles depending on the different electrical properties of the particles. Under DEP, the smaller DNA accumulated in the DEP high-field regions while the bigger blood cells were concentrated into the DEP low-field regions due to their different polarizability. The results showed that CLL patients but not healthy controls have a considerable amount of SYBR Green stained DNA concentrated in the DEP high-field regions. The expression profile of microRNAs (miRNA) can also be used to detect different cancers, making it an important target in cancer research. However, detection of circulating miRNA is challenged by its extremely low concentration in human body fluid. Arata et al. developed a power-free microfluidic device to directly detect miRNA in a rapid and sensitive manner without PCR amplification (Figure 3C) [24]. This detection is based on the hybridization process, where a target nucleic acid can bind to its complementary oligonucleotide capture and detection probes. The miRNA was detected by a sandwich hybridization method, in which the signal was amplified by laminar flow-assisted dendritic amplification. The platform used the degassed PDMS to drive the liquid, thus eliminating the need of external pressure sources. The device can detect miRNA-21 at the LOD of 0.5 pM from 0.5 mL of sample within 20 min [55]. Simultaneous detection of multiple nuclei features in individual cells is an important technique for cancer cell identification, and useful for minimal residual disease (MRD) analysis. Mughal et al. developed a simple microfluidic-based fluorescence in-situ hybridization (FISH) assay to screen hematopoietic malignancies [28]. The device was fabricated by photolithography and etching on a glass slide. Cells were immobilized in the microchannels and were used for FISH analysis. This device can analyze 10 samples in sub-microliter volume with the LOD of 1:100 cells in MRD analysis, which is lower than the 1:1000 cells from the conventional FISH.

### 3.3. Detection of Exosomes as Cancer Biomarkers

Compared with circulating proteins and nucleic acids in body fluids, exosomes are emerging as a new and attractive class of cancer biomarkers [56]. An exosome is a nano-sized vesicle secreted by cells in all body fluids and contains cell-specific molecular and genetic content. Exosomes play an important role in the pathological processes of cancer through intercellular communication. Due to both their presence in body fluids and resemblance of their contents to parental cells, exosomes are considered liquid biopsy specimens. The major hurdle in the utilization of exosomes has been the lack of a consistent isolation method. The conventional methods include ultracentrifugation, ultrafiltration and affinity capture, but these methods are time-consuming and suffer from low recovery yield and low specificity. LoC techniques have shown potential to improve recovery rate, reduce the sample size, and enhance exosome detection [57]. For example, He et al. developed a microfluidic device to enable sequence immunoisolation and protein analysis of circulating exosomes within ~100 min using only 30 μL of plasma samples [25]. They successfully demonstrated this method to assess the total expression and phosphorylation levels of insulin-like growth factor 1 receptor (IGF-1R) in non-small-cell lung cancer patients. In another study, Zhao et al. used a continuous-flow microfluidic device (ExoSearch) to quantitatively isolate and release the exosomes from blood plasma [26]. Using this device, they performed a multiplexed measurement of three exosomal tumor markers, including cancer antigen 125 (CA-125), epithelial cell adhesion molecule (EpCAM), and CD24, from the plasma of an ovarian cancer patient.

### 3.4. Detection of Circulating Tumor Cells (CTC) as Cancer Biomarkers

In addition to protein and genetic biomarkers for cancer diagnosis, isolation and detection of CTC has drawn significant attention over recent years [58,59,60]. LoC technologies have been increasingly developed to improve CTC-based diagnosis [61,62,63]. The main challenge of CTC detection is the extremely low CTC number in blood. In this regard, different LoC devices were developed to trap CTC directly from whole blood based on both the surface protein marker and morphological properties of CTC [64,65]. To enhance CTC trapping, different microfluidic device designs were developed [30,66,67] such as chaotic mixing modules, fluidic separations and mechanical traps (Figure 3D) [30]. In addition, CTC detection in miniaturized devices requires the ability to efficiently process a relatively large volume of blood sample on a chip, which has been an area of important research and development [68,69]. Given the vast literature in this field, we refer the readers to a number of excellent reviews for more details [70,71,72,73,74,75,76,77,78,79,80,81,82,83].

In summary, LoC-based platforms offer new tools for detecting various types of cancer biomarkers such as genetic biomarkers, proteins and cells. Fast test speed, multiplexing, and the ability to detect rare events are the attractive features of LoC platforms for cancer diagnosis.

## 4. Conclusions and Future Perspectives

Early diagnosis of CVD and cancer is critical for successful treatment and recovery. Development of rapid, simple and sensitive diagnostic methods that can detect multiple biomarkers in biological fluids for CVD and cancer diagnosis is highly desirable. In this direction, LoC-based biosensors have shown great potential to enable such advanced diagnostic applications as reviewed in this paper.

Looking forward, the challenges of LoC-based diagnosis for CVD and cancer also present exciting opportunities. For example, both CVD and cancer share biomarkers with other inflammatory diseases. It is difficult to distinguish these diseases based on single biomarkers. Multi-biomarker analysis is an attractive approach to improve the diagnosis accuracy. Compared with other circulating biomarkers, exosomes provide an ideal resource for specific multi-biomarker detection as they contain the fingerprint information of the releasing cells. LoC platforms not only address the purification issue of traditional methods, but also enable rapid proteomics or genetic analysis in the same platform. The biological function of exosomes is still not completely clear and further research enabled by LoC technology will generate important insights. Furthermore, the LoC-based multiplex testing has the potential to identify new biomarkers by proteomic, genetic and metabolic profiling of different biofluids.

Nanomaterials have been increasingly integrated into bioanalytical devices since they can improve the detection sensitivity and detection limits to single molecules. Nano particles have been widely used due to their easy synthesis and easy integration with sensors. Although other nanomaterials such as nanowire and nanotubes have unique physical and chemical characteristics as biosensors, the major challenge is their complex fabrication and the difficulty in interpreting the measurements. Further efforts are required to improve fabrication and characterizations of these nanomaterials.

Recently, there has been a growing trend to use smartphones as the detection tools to read the signals from the LoC biosensors. We called this the MS^2^ technology (i.e., mobile sensing based on microfluidic devices and smartphone), which takes advantage of the powerful imaging and computing capabilities of smartphones to achieve rapid data acquisition and reporting [84]. It not only facilitates quantitative diagnosis but also enables remote communication, which is attractive for PoC monitoring of both CVD and cancer.

Besides the molecular biomarkers, cell-based tests can provide additional valuable information for the diagnosis of CVD and cancer. On the other hand, cell-based tests require more complicated LoC platforms, which need to be addressed by advanced system integration. Among the cell-based tests, the specificity of using WBC and EPC numbers as CVD biomarkers needs further validation. In addition, further technology development towards efficient CTC purification from large volumes of blood and the integration of downstream molecular analysis in the LoC platform will benefit cancer diagnosis.

## Figures and Tables

**Table 1:** Summary of examples LoC-based platforms for CVD and cancer biomarkers detection. Note: a list of abbreviations has been provided at the end of the article.

Disease	Marker	Technique	LOD	Detector	Assay Time	Sample Volume	Ref.
**CVD**	hs-CRP	bead-based ELISA	0.30 ng/mL (saliva); 0.27 ng/mL (blood)	CCD	<20 min	200 μL	[16]
	cTnI		0.51 ng/mL (saliva); 0.27 ng/mL (blood)				
	NT-proBNP		0.24 ng/mL (saliva); 0.32 ng/mL (blood)				
	myoglobin	aptamer-antibody hybrid assay	-	Blu-ray optical drive	-	-	[17]
	troponin I						
	CRP						
	CK-MB	surface acoustic wave	<1 nM	vector network analyzer	<30 min	-	[18]
	D-dimer						
	PAPP-A						
	CRP	fluorescence	0.54 μg/mL	fluorescent microscope	5 min	~4.5 μL	[19]
	WBC	colorimetric assays	-	naked eye	~35 min	15 μL	[20]
	EPC	immunofluorescence	-	fluorescent microscope	1 h	200 μL	[21]
**Cancer**	PSA	ECL	300–500 fg/mL	CCD	35 min	2–5 μL	[22]
	PSMA						
	PF-4						
	CA125	bead-based immunoassay	1.8 U/mL	fluorescent microscope	43 min	50 μL	[23]
	HE4		2.3 pmol/L				
	MMP-7		0.2 ng/mL				
	CA72-4		1.7 U/mL				
	miRNA-21	sandwich hybridization	0.5 pmol/L	fluorescent microscope	20 min	500 μL	[24]
	IGF-1R	Exosome immunoisolation and protein analysis	0.281 pg/mL	fluorescent microscope	~100 min	30 μL	[25]
	p-IGF-1R		0.383 pg/mL				
	CA-125	immunomagnetic beads exosome isolation and immunofluorescence protein analysis	-	fluorescent microscope	40 min	20 μL	[26]
	EpCAM						
	CD24						
	cfc-DNA	DEP isolation	8–16 ng/mL	fluorescent microscope	-	20 μL	[27]
	cancer cell	FISH	1:100 (MRD)	microscope	1 h	0.2 μL	[28]
	KRAS	digital PCR	0.05–0.1% (mutant alleles)	real-time PCR	~1 h	1.8 μL	[29]
	CTC	lateral migration	-	a microscope equipped with a high-speed camera	~2 min	1 mL	[30]

## Abbreviations

3D	three-dimensional
CA	carbohydrate antigen
CF	cystic fibrosis
Cfc	cell free circulating
CK-MB	creatine kinase MB
CLL	chronic lymphocytic leukemia
CRP	C-reactive protein
CTC	circulating tumor cell
CTI	cleavable tag immunoassay
cTn	cardiac troponin
CVD	cardiovascular diseases
DEP	dielectrophoretic
ECL	electrochemiluminescent
EGF	epidermal growth factor
ELISA	enzyme linked immunosorbent assay
EPC	endothelial progenitor cells
FETs	field-effect transistors
FISH	fluorescence in-situ hybridization
HDL3	high density lipoprotein-3
HE4	human epididymis protein 4
hs-CRP	high-sensitivity C-reactive protein
LoC	lab-on-a-chip
LOD	limit of detection
MMP	matrix metalloproteinase
MRD	minimal residual disease
MRI	magnetic resonance imaging
MS	mass spectrometry
MS^2^	mobile sensing based on microfluidic devices and smartphone
NT-proBNP	N-terminal pro-B type natriuretic peptide
PAI-1	plasminogen activator inhibitor-1
PCR	polymerase chain reaction
PDMS	polydimethylsiloxane
pM	picomolar
PSA	prostate specific antigen
PSMA	prostate specific membrane antigen
qPCR	quantitative polymerase chain reaction
RBC	red blood cell
SAW	surface acoustic wave
WBC	white blood cells
WHO	World Health Organization
